# Preoperative Germline Genetic Testing and Surgical Timing in Breast Cancer: Implementation of National Reimbursement Programs: A Retrospective Cohort Study

**DOI:** 10.3390/medicina62071397

**Published:** 2026-07-19

**Authors:** Ioan-Catalin Vlad, Constantin-Iulian Vlad, Ovidiu Balacescu, Vlad-Alexandru Gata, Dragos-Stefan Morariu, Maria Miclaus, Ana Lucia Muntean, Andreea Catana, Nicoleta Antone, Patriciu Achimas-Cadariu

**Affiliations:** 1 Department of Oncology, University of Medicine and Pharmacy “Iuliu Hațieganu”, 400347 Cluj-Napoca, Romania; 2 Department of Surgical Oncology, The Oncology Institute “Prof. Dr. Ion Chiricuță”, 400015 Cluj-Napoca, Romania; 3 Department of Genetics, Genomics and Experimental Pathology, The Oncology Institute “Prof. Dr. Ion Chiricuță”, 400015 Cluj-Napoca, Romania; 4 Department of Oncogenetics, The Oncology Institute “Prof. Dr. Ion Chiricuță”, 400015 Cluj-Napoca, Romania; 5 Department of Medical Education, University of Medicine and Pharmacy “Iuliu Hațieganu”, 400347 Cluj-Napoca, Romania; 6 Department of Molecular Sciences, University of Medicine and Pharmacy “Iuliu Hațieganu”, 400347 Cluj-Napoca, Romania; 7 Department of Medical Oncology, The Oncology Institute “Prof. Dr. Ion Chiricuță”, 400015 Cluj-Napoca, Romania

**Keywords:** breast cancer, genetic testing, BRCA1, BRCA2, surgical timing, neoadjuvant systemic therapy, surgical workflow, precision oncology

## Abstract

*Background and Objectives*: Over recent decades, germline genetic testing has become increasingly integrated into breast cancer care, yet its precise effect on the timing of surgical workflows remains incompletely defined. This study investigates the implementation of nationally reimbursed genetic testing programs and examines how the scheduling of multigene germline testing relates to surgical timing. *Materials and Methods*: In this retrospective cohort analysis, we examined 502 breast cancer cases from the institutional registry of the “Ion Chiricuță” Oncology Institute (IOCN), with a mean age of 52 years, a high rate of neoadjuvant therapy (82.3%) and a majority of them in T2 (52%) and N1 (43%), N2 (29%) clinical stage: 263 patients from an earlier private-practice pay testing era (pre-reimbursement) as a comparison sample and 239 patients from the period when reimbursement programs were in operation. We then evaluated the benefits of state-funded genetic testing initiatives, computing three intervals: diagnosis to genetic test, genetic test to surgery, and diagnosis to surgery. Patients were categorized by timing of multigene testing (preoperative vs. postoperative), receipt of neoadjuvant systemic therapy (NACT), mutation status, and funding source for testing (national PNS program funded by the Romanian Ministry of Health; PNRR European Recovery and Resilience Facility funds; or self-funded private testing). Nonparametric statistics (Mann–Whitney U, Spearman correlation) and effect-size metrics (Cliff’s delta, Theil–Sen slope) were employed. *Results*: The median diagnosis-to-surgery interval was 203 days (IQR 179–230). Patients tested preoperatively had longer intervals than those tested postoperatively (216 vs. 182.5 days; *p* = 0.000153; Cliff’s δ = −0.486), a pattern driven by shared pathways involving NACT rather than by testing-induced delays. NACT was the principal determinant of surgical timing (211 vs. 43 days; *p* = 302.55 × 10^−11^). Within the preoperative subgroup, time to multigene testing correlated strongly with time to surgery (Spearman ρ = 0.54; *p* = 4.75 × 10^−7^; Theil–Sen slope = 0.37, 95% CI = 0.22–0.53). However, the no-NACT group included only four patients. The presence of a pathogenic variant did not significantly change surgical timing (*p* = 0.982). The PNS national reimbursement program achieved the highest preoperative integration rate for multigene genetic testing after adjustment for NACT confounder (76.7%), outperforming PNRR (61%) and private practice (56%). *Conclusions*: While genetic testing timing correlates with surgical workflow, neoadjuvant systemic therapy pathways and program structure exert greater influence than testing per se. Structured national programs enhance preoperative testing uptake without causing delays beyond those inherent to NACT pathways.

## 1. Introduction

Breast cancer remains the most frequently diagnosed malignancy among women worldwide, with approximately 2.3 million new cases annually [1]. The significance of high-penetrance susceptibility genes, identified through multigene genetic testing—particularly BRCA1 and BRCA2—has transformed clinical practice by facilitating individualized risk assessment, cascade testing, and customized treatment strategies [2,3]. The BRCA1 and BRCA2 susceptibility genes are tumor suppressor genes; in their pathogenic or potentially pathogenic variants, they are clinically associated with hereditary breast and ovarian cancer syndrome, which follows an autosomal dominant pattern of inheritance [4]. Pathogenic variants in BRCA 1/2 are associated with a cumulative lifetime breast cancer risk of 60–80%, markedly surpassing the general population risk of approximately 12% [5,6].

In addition to risk assessment, BRCA1/2 mutation status has become a predictive marker with direct therapeutic implications, including eligibility for poly(ADP-ribose) polymerase (PARP) inhibitors in both early-stage and metastatic disease, establishing multigene genetic testing as a vital component of treatment planning [7,8,9,10,11]. Current international guidelines support broader implementation of germline genetic testing in breast cancer. Recommendations from the European Society for Medical Oncology (ESMO) and the National Comprehensive Cancer Network (NCCN) include testing for patients with triple-negative breast cancer, those diagnosed before age 50, and individuals with family histories suggestive of hereditary cancer syndromes [12,13]. The American Society of Breast Surgeons recommends offering genetic testing to all patients diagnosed with breast cancer [14], while recent ASCO recommendations support broader eligibility criteria, including testing up to 65 years of age in selected settings [15]. In accordance with these evolving recommendations, our institutional protocol recommends germline genetic testing for all breast cancer patients younger than 65 years.

Beyond systemic treatment implications, the timing of germline genetic testing has become increasingly important for surgical decision-making. Availability of preoperative genetic results may influence the extent of surgery, including decisions regarding breast-conserving surgery, unilateral or bilateral mastectomy, contralateral risk-reducing procedures, and reconstructive planning [13,14,15,16]. Consequently, delayed access to testing may limit the integration of genetic information into the initial treatment pathway.

Genetic testing prior to surgery in patients newly diagnosed with early-stage invasive breast cancer has been found to significantly influence the surgical approach; those with pathogenic variants in BRCA, PALB2, and TP53 are recommended to undergo prophylactic contralateral mastectomy during the same surgical procedure [17,18,19]. In a retrospective study, the benefits of this recommendation have been demonstrated by showing a reduction in breast cancer risk of over 90% among high-risk women who opted for post-test prophylactic bilateral mastectomy [20]. Another study reveals that, following genetic testing and risk classification, the choice of prophylactic mastectomy increased life expectancy by approximately 5 years in women with an estimated risk of 85% [21].

The attending surgeon accounted for about 20% of the variation in receipt of contralateral prophylactic mastectomy (CPM) after controlling for patient factors, indicating substantial surgeon-level effects on practice. Physician attitudes influence both willingness to offer CPM and patients’ eventual choices, with patients being nearly three times more likely to receive CPM if they saw a surgeon whose practice had a higher-than-average CPM rate (OR 2.8 per 1 SD increase in surgeon CPM tendency) [22,23].

Despite the established clinical utility of genetic testing, the optimal timing of testing during the treatment course remains an area of ongoing research and exhibits considerable variability in practice [16]. Preoperative genetic testing has the potential to inform initial surgical planning, possibly reducing the need for subsequent risk-reducing procedures and enabling patients to make fully informed decisions regarding bilateral mastectomy, contralateral prophylactic surgery, and prophylactic Bilateral Salpingo-Oophorectomy (BSO) [24,25].

Women who carry BRCA1/2 susceptibility mutations are at risk of developing both breast and ovarian cancer. In the case of ovarian cancer, the risk ranges from 20 to 40 percent. Given this increased risk—which is 10 times higher than that of the general population—the recommendation for salpingo-oophorectomy appears reasonable [26]. Furthermore, the lack of effective and early diagnostic measures means that most forms of ovarian cancer are detected at advanced stages, when the disease is highly aggressive and has a poor prognosis, making the case for prophylactic surgery even stronger [18,27,28]. In this case, parity, the desire to have more children, and family history should be taken into account [13]. Reproductive options are also a sensitive issue following a positive genetic test result for a PV or LPV breast cancer susceptibility gene. Prenatal genetic diagnosis or assisted reproduction with preimplantation genetic testing may be offered as solutions [13,27]. Assisted reproductive technologies, which involve collecting and preserving many oocytes before gonadotoxic therapies combined with family planning, have enabled pregnancy rates in PV mutation patients with early-stage invasive breast cancer to match those of the general population [29].

Despite what has been presented, obtaining genetic test results prior to surgery may introduce delays in definitive local treatment, especially in healthcare systems where rapid testing infrastructure is not universally available [30]. Quantifying this potential delay—and identifying the patient populations and systemic factors that contribute to it—is crucial for designing workflows that maintain the informational advantages of preoperative testing without compromising the timeliness of surgical intervention.

Neoadjuvant systemic therapy (NACT) has become the standard of care for locally advanced breast cancer and is increasingly used in earlier-stage disease, particularly in triple-negative and HER2-positive subtypes [31,32,33,34,35]. The neoadjuvant treatment window—typically consisting of 4–6 months—provides a practical opportunity to complete genetic testing and counseling without delaying systemic therapy [1,12].

The interplay among genetic testing timing, neoadjuvant systemic therapy pathways, and surgical workflow has not been comprehensively characterized in real-world cohorts, particularly across different healthcare funding models. Access to funded germline testing varies considerably across healthcare systems, and the introduction of national reimbursement policies has the potential to standardize testing pathways, reduce out-of-pocket barriers, and—if implemented with appropriate infrastructure—improve the timeliness of result delivery [16,25]. Conversely, poorly coordinated reimbursement frameworks may inadvertently create administrative bottlenecks that prolong the diagnosis-to-surgery interval. Understanding whether national funding programs can improve the integration of genetic testing without adding delays to care is therefore critical for healthcare policy and clinical pathway design.

In this context, the present study aimed to evaluate the real-world relationship of implementing national reimbursement programs for germline genetic testing on surgical workflow in breast cancer patients treated at the Oncology Institute “Prof. Dr. Ion Chiricuță”, Cluj-Napoca, Romania. Using a retrospective cohort of 502 breast cancer patients from a Romanian oncological center, we examined: (1) whether genetic testing timing (preoperative vs. postoperative) is independently associated with the diagnosis-to-surgery interval; (2) the relative contribution of NACT and program structure to this interval; (4) whether pathogenic germline variant status (positive versus negative) influences surgical planning; and (5) how multigene panel testing is integrated across different healthcare reimbursement programs for genetic testing.

## 2. Materials and Methods

### 2.1. Study Design and Patient Population

This retrospective cohort study examined a dataset comprising 502 breast cancer patients from the “Ion Chiricuță” Oncology Institute Cluj-Napoca, Romania who underwent multigene germline genetic testing. The first cohort, analysed from March 2024, consisted of 263 patients from the IOCN genetics department, from the out-of-pocket era (tested between July 2017 and March 2024), in which genetic testing was conducted by private practices and served as the reference point for our comparative analysis. The second cohort, analysed from March 2026, included 239 patients tested between January 2023 and March 2026, a period corresponding to the implementation of various programs (European-funded PNRR, the governmental National Health Program-PNS) within our institution, primarily analyzed to obtain pertinent data on their implementation.

**Figure 1:**
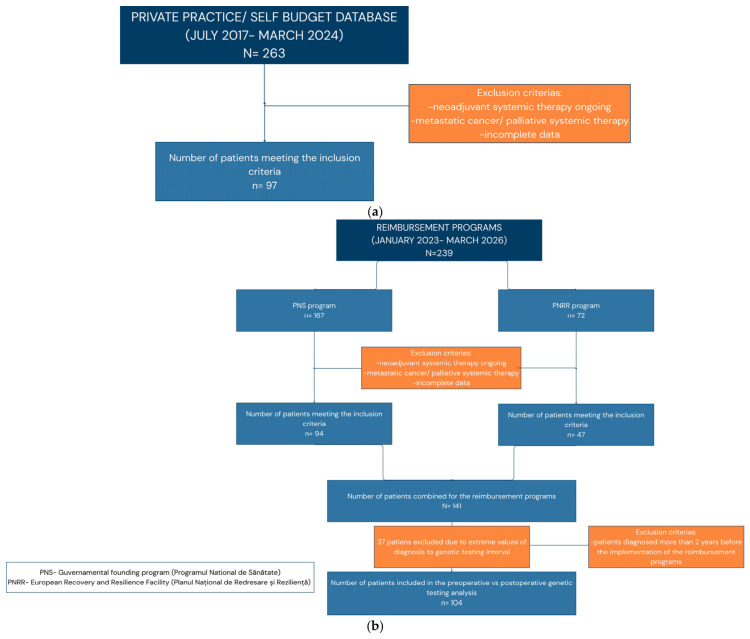
(**a**). STROBE Flow Diagram of the pre-reimbursement period cohort. (**b**). STROBE Flow Diagram of the reimbursement programs cohort.

All patients included had a pathological confirmation of breast cancer and underwent germline genetic testing via our institution, utilizing next-generation sequencing (NGS) multigene panels that targeted hereditary breast cancer susceptibility genes. All panels included BRCA1 and BRCA2 as core genes, together with other clinically relevant breast cancer susceptibility genes such as CHEK2, PALB2, and TP53. Germline genetic testing was performed for all eligible patients according to institutional criteria. Patients with incomplete or unavailable medical records due to missing information in the patients’ files or inaccessible external/territorial medical or surgical treatments, those who had not yet completed neoadjuvant systemic therapy (NACT) at the time of data collection, and patients with metastatic disease receiving palliative systemic therapy were excluded, as these situations were deemed to interfere with the selected statistical analysis intervals. The STROBE diagram in Figure 1a,b depicts the cohort after applying these criteria.

**Figure 2:**
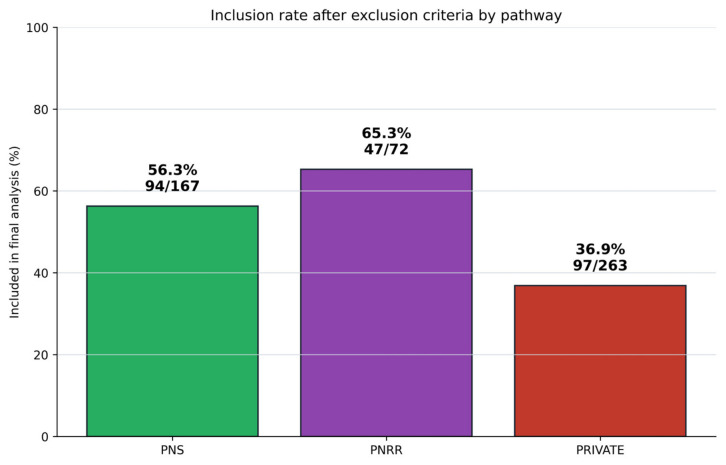
Proportion of patients included in each program after applying the exclusion criteria.

To assess potential selection bias, we compared the proportion of patients retained in the final analysis across funding pathways. Inclusion rates differed significantly across groups: 36.9% in private practice, 56.3% in PNS, and 65.3% in PNRR (χ^2^ *p* = 0.000002). Private-practice patients were significantly less likely to be included compared with PNS (OR 0.45, 95% CI 0.31–0.67, *p* = 0.000099) and PNRR (OR 0.31, 95% CI 0.18–0.54, *p* = 0.000025), whereas inclusion rates did not significantly differ between PNS and PNRR (*p* = 0.201) (Figure 2).

### 2.2. Data Collection

Clinical and demographic data were extracted from electronic medical records. The following variables were collected: age at diagnosis; date of pathological diagnosis (defined as the date of biopsy confirming invasive breast carcinoma or ductal carcinoma in situ); date of germline genetic testing sample collection; date of definitive surgical intervention; date of administration of neoadjuvant systemic therapy (NACT); results of genetic testing (classified as pathogenic variant positive, negative, or variant of uncertain significance); and funding source (PNS national programme, PNRR European programme, or self-funded testing through private providers).

### 2.3. Endpoint Definitions

The primary endpoint was the diagnosis-to-surgery interval, defined as the number of days between histopathological confirmation of breast cancer and definitive surgery. Secondary endpoints included the proportion of patients receiving preoperative versus postoperative genetic testing, the influence of NACT on testing integration, and differences between reimbursement pathways. Genetic testing was classified as preoperative when genetic test results were available before definitive surgery and postoperative when results became available after surgery.

### 2.4. Healthcare Program Definitions

Patients were assigned to a reimbursement healthcare pathway according to the program available at the time of genetic testing; when reimbursement was unavailable, they were assigned to the self-funded testing pathway. The PNS (Programul Național de Sănătate) is a government initiative, funded by the Romanian Ministry of Health, that provides structured access to BRCA1/2 genetic testing, with specific eligibility criteria, dedicated genetic counseling slots, and mandatory documentation. This program was implemented in Romania during 2022–2023 as part of the National Cancer Control Plan. The PNRR (Planul Național de Redresare și Reziliență), funded through the National Recovery and Resilience Facility starting in November 2021, is an innovative initiative aimed at subsidizing and developing the genetic department within our institution. It expanded access to testing, but with less structured referral pathways and without the mandatory documentation framework of the PNS. The private practice group comprises self-funded patients who met testing criteria and were referred to external laboratories, often paying out of pocket before reimbursement programs were established. This cohort serves as a referral comparison group. These three models differ in eligibility criteria, referral mechanisms, and the degree of pathway standardization, which are hypothesized to explain observed differences in preoperative testing rates.

To assess the comparative efficacy of these programs in clinical practice, data from 263 patients tested in the private practice setting were incorporated into our reimbursement cohort database and analyzed using the same statistical indicators to ensure relevance.

### 2.5. Key Time Intervals

Three primary time intervals were computed for each patient: (1) Diagnosis to genetic testing (Dx→GT): the duration in days from histopathological diagnosis to the collection of the genetic testing sample; (2) Genetic testing to surgery (GT→Surgery): the duration in days from genetic testing to definitive surgery—negative values indicate that testing was conducted after surgery; (3) Diagnosis to surgery (Dx→Surgery): the duration in days from histopathological diagnosis to definitive surgery. Patients were categorized into two principal groups based on the timing of genetic testing: preoperative genetic testing (GT→Surgery > 0 days) and postoperative genetic testing (GT→Surgery < 0 days).

### 2.6. Statistical Analysis

Continuous variables were summarized using medians and interquartile ranges (IQR), owing to non-normal distributions confirmed through the Shapiro–Wilk test. Comparisons between two groups were conducted using the Mann–Whitney U test, while the Kruskal–Wallis test was employed for three or more groups, with post hoc pairwise comparisons utilizing Dunn’s test. Effect sizes for two-group comparisons were measured using Cliff’s delta (δ). The correlation between continuous time variables was examined via Spearman’s rank correlation coefficient (ρ), with the Theil–Sen estimator applied for robust slope estimation. All statistical analyses were two-tailed; significance was established at *p* < 0.05. Analyses were performed using Python 3.11 (SciPy 1.11, pandas 2.0).

### 2.7. Ethical Aspects

The study received approval from the Ethics Committee of the Oncological Institute “Ion Chiricuță” in Cluj-Napoca (approval number 288/5 March 2024), in accordance with the study protocol and aligned with national and international standards.

Informed consent was waived by the Ethics Committee due to the retrospective, non-interventional nature of the study.

All relevant regulations concerning confidentiality and the use of personal data were adhered to.

## 3. Results

### 3.1. Program Comparison: Integration of Preoperative Genetic Testing in the Main Cohort

Following the application of inclusion criteria for the two patient databases, data from those with complete information were analyzed and compared as follows: a total of 94 patients from the 167 beneficiaries of the PNS program, 47 out of 72 from the PNRR program, and 97 out of 263 tested in the self-funded group, as depicted in Figure 1a,b.

**Figure 3:**
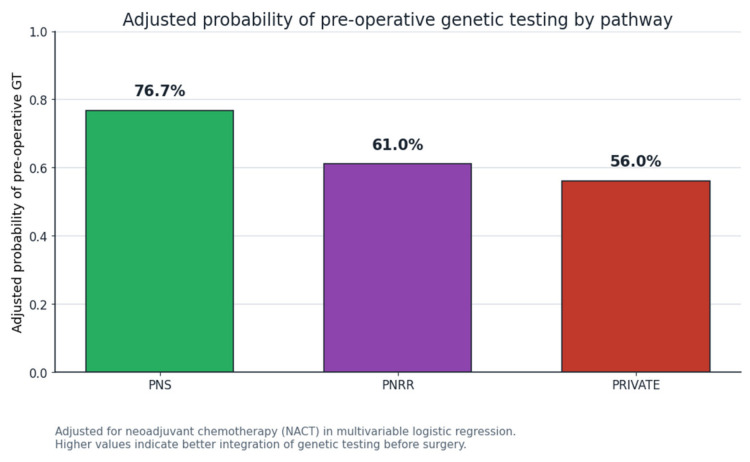
Comparison between the efficacy of the different founding sources for genetic testing regarding the integration of the tests in the clinical pathway, after adjusting NACT cofounder, for all programs.

Given that neoadjuvant chemotherapy constitutes a major confounding factor—substantially prolonging the diagnosis-to-surgery interval and being unequally distributed across programs—a multivariable logistic regression adjusted for NACT was performed to estimate the adjusted probability of preoperative genetic testing integration for each program. The PNS program served as the reference group. The analysis indicated that the PNS program remained associated with the highest probability of preoperative genetic testing. After adjustment for neoadjuvant chemotherapy, the PNS program exhibited the highest adjusted probability of preoperative genetic testing (76.7%), compared with PNRR (61.0%) and private-practice testing (56.0%) (see Table 1, Figure 3). Private-practice testing demonstrated significantly lower adjusted odds of preoperative testing compared with the PNS program (OR 0.39, 95% CI 0.19–0.80, *p* = 0.0097), thereby supporting the superior preoperative integration of genetic testing within the PNS pathway, as detailed in Table 2.

The interval from diagnosis to genetic testing did not differ significantly across programs (PNRR median 135 days, PNS 162 days, private practice 159 days; all pairwise *p* > 0.5). Nonetheless, the duration from diagnosis to surgical intervention, encompassing the entire pathway including neoadjuvant systemic therapy, case evaluation by the Tumor Board Committee, and administrative processes, was notably shorter for private practice patients than for those in the PNS group, with a difference of 28.0 days (*p* = 0.0031) in the ordinary least-squares (OLS) linear regression and a smaller difference of 14 days (*p* = 0.0031) in the robust median regression (refer to Table 3 and Table 4).

In adjusted models, neoadjuvant chemotherapy was the strongest determinant of time to surgery, increasing the diagnosis-to-surgery interval by approximately 147 days in OLS regression and 161 days in median regression. Program type had a smaller effect on surgical timing than NACT. Private-practice testing was associated with a shorter adjusted mean time to surgery than PNS, but this association was attenuated in median regression, suggesting sensitivity to distributional differences and outliers.

### 3.2. Analysis for Reimbursement Cohort

This study included 239 breast cancer patients who underwent germline genetic testing during the reimbursement program period, between January 2023 and March 2026. Among patients with complete timing data who met inclusion criteria (*n* = 141), the mean age at diagnosis was 52 years. All analyzed patients were alive at the last follow-up and had no metastases, as metastases were an exclusion criterion. A brief clinical description of these patients’ characteristics can be found in Table 5.

**Figure 4:**
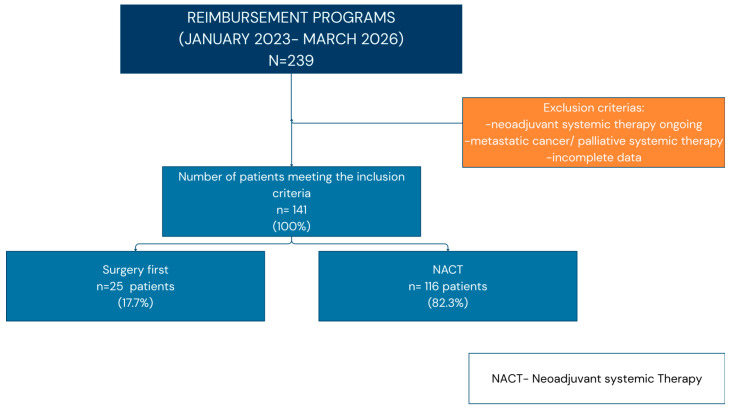
Analysis of clinical pathways in the reimbursement programs cohort.

The majority received neoadjuvant systemic therapy (NACT) (n = 116; 82.3%), reflecting the predominance of locally advanced disease within this cohort. Twenty-five patients (17.7%) underwent surgery first, as illustrated in Figure 4.

**Figure 5:**
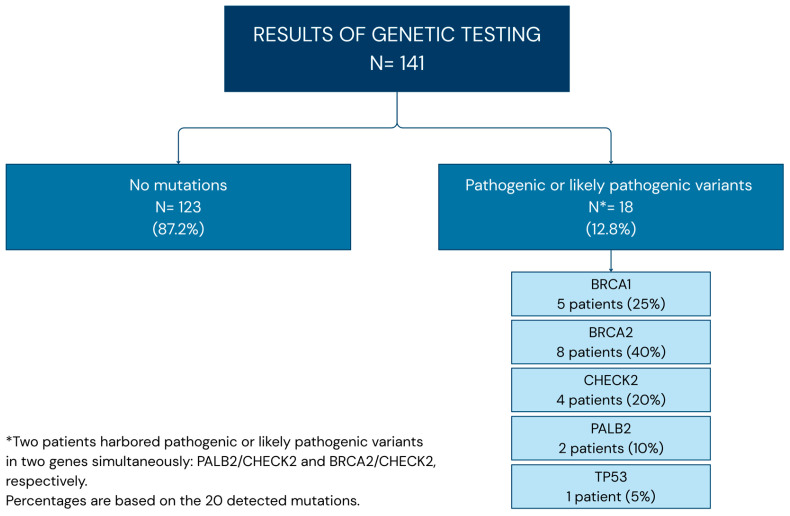
Results of genetic testing in the reimbursement cohort.

Genetic testing revealed pathogenic or likely pathogenic variants in 18 patients (12.8%), of whom two were carriers of mutations in two genes. The distribution of these genetic variants is illustrated in Figure 5.

### 3.3. Overall Time from Diagnosis to Surgery in the Reimbursement Study Cohort

**Figure 6:**
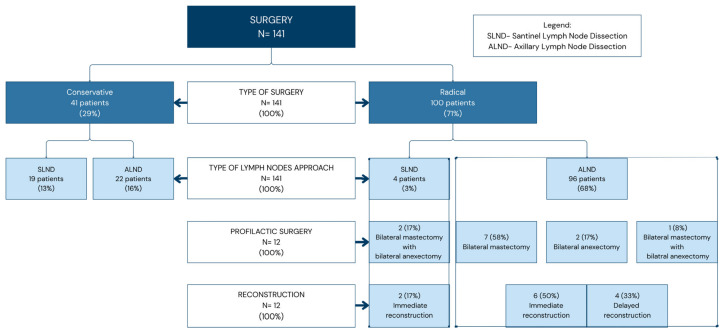
Analysis of the surgical approaches in the reimbursement programs cohort.

**Figure 7:**
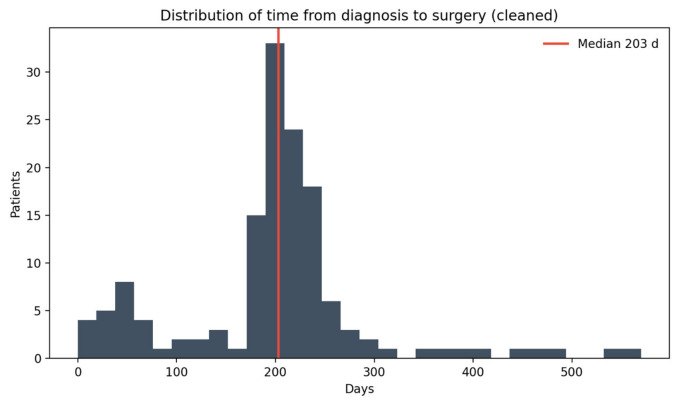
Time from diagnosis to surgery in the reimbursement programs cohort.

Among the 141 patients undergoing surgery, as depicted in Figure 6, the median duration from pathological diagnosis to definitive surgery was 203 days (Interquartile Range 179–230 days; mean 198.3 ± 96.9 days), as illustrated in Figure 7. The distribution was concentrated around the six-to-seven-month period, which aligns with the substantial proportion of patients completing comprehensive Neoadjuvant Systemic therapy (NACT) pathways. Additionally, this timeframe encompasses tumor board evaluations and administrative processes (see Table 6).

Time from diagnosis to surgery and from diagnosis to genetic testing were compared across mutation types. No statistically significant differences were observed across the main evaluable groups (Negative, BRCA1, BRCA2) for either the diagnosis-to-surgery interval (Kruskal–Wallis *p* = 0.347) or the diagnosis-to-genetic testing interval (*p* = 0.211). Mutation-specific analyses were limited by small subgroup sizes, particularly for CHEK2, PALB2, TP53, and multiple-gene alterations, and should therefore be interpreted as exploratory.

### 3.4. Preoperative Versus Postoperative Genetic Testing Comparison in the Reimbursement Study Cohort

**Figure 8:**
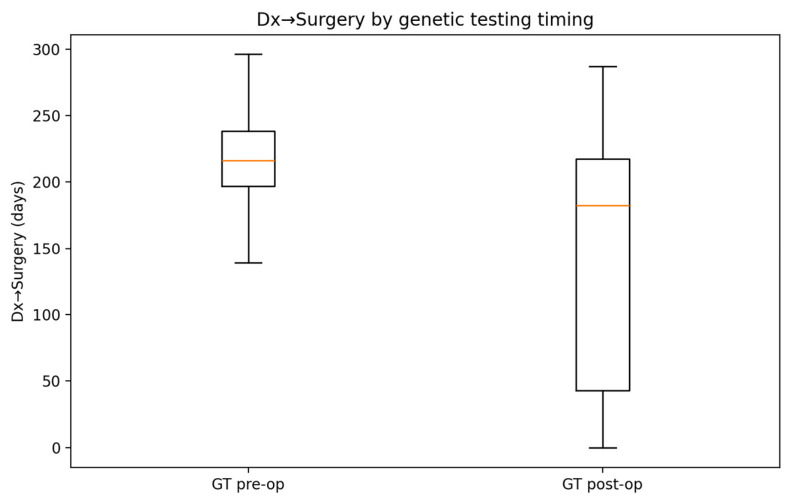
Comparison between the time span from diagnosis (Dx) to surgery in the preoperative versus postoperative genetic testing (GT) groups in the reimbursement study cohort.

Among 104 patients with comprehensive data for this section (exclusion criteria: diagnosis more than 2 years before the implementation of the reimbursement programs, as indicated in Figure 1a), 76 (73.1%) underwent genetic testing prior to surgery (preoperative group), while 28 (26.9%) received testing subsequent to surgery (postoperative group). Patients in the preoperative group exhibited significantly longer intervals from diagnosis to surgery, with a median of 216 days (IQR 197–238), compared to 182.5 days (IQR 42.8–217.2) in the postoperative group (Mann–Whitney U test, *p* = 0.000153; Cliff’s δ = −0.486, indicating a moderate-to-large effect size) (see Table 7, Figure 8). This pattern mirrors the dynamics of clinical workflows: patients who proceed expeditiously to surgery tend to have genetic testing ordered after the operation, whereas those undergoing longer treatment courses, particularly with neoadjuvant systemic therapy (NACT), tend to have genetic testing results available prior to surgery [24,25].

### 3.5. Neoadjuvant Systemic Therapy: One of the Determinants of Surgical Timing in the Reimbursement Study Cohort

**Figure 9:**
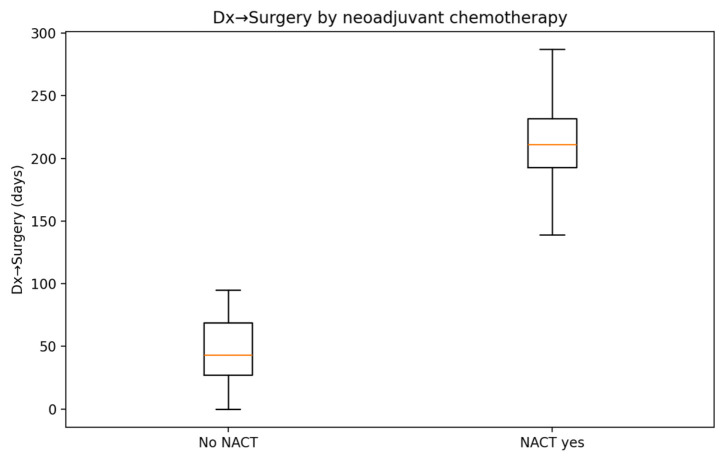
Comparison between the time span from diagnosis to surgery in the group of patients who received neoadjuvant systemic therapy (NACT) versus those who did not.

Neoadjuvant systemic therapy significantly influenced the time from diagnosis to surgery. Among 141 patients, 116 (82.3%) received NACT, while 25 (17.7%) did not. The median interval from diagnosis to surgery was 211 days (IQR 193–232) for those who received NACT, compared to just 43 days (IQR 27–69) for patients who proceeded directly to surgery (Mann–Whitney U test, *p* = 2.55 × 10^−11^; Cliff’s δ = 0.92, indicating a large effect size) (Table 8, Figure 9). In the non-NACT group, only 4 patients underwent preoperative genetic testing, making it difficult to evaluate any testing-related delays within this fast-track group.

### 3.6. Correlation Between Genetic Testing Timing and Surgical Timing in the Reimbursement Study Cohort

**Figure 10:**
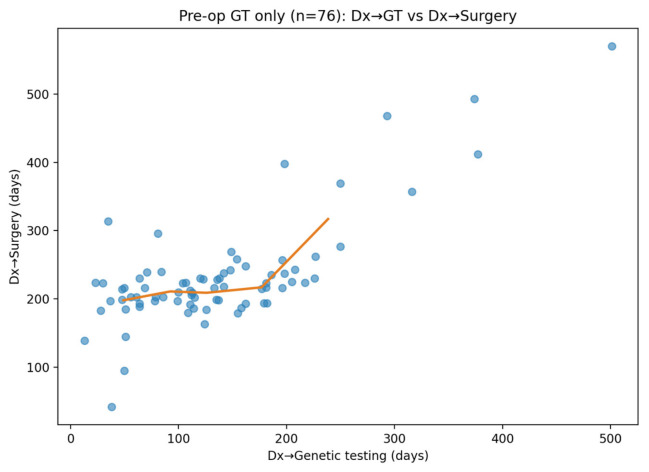
Correlation between the time from diagnosis (Dx) to genetic testing (GT) and the one from diagnosis to surgery in the reimbursement stud cohort.

Among the 76 patients who underwent genetic testing prior to surgery, a prolonged interval from diagnosis to genetic testing—Dx→GT—was strongly correlated with an extended duration from diagnosis to surgical intervention—Dx→Surgery (Spearman ρ = 0.54; *p* = 4.75 × 10^−7^). The Theil–Sen robust slope estimate indicated that each additional day from diagnosis to genetic testing corresponded to approximately 0.37 additional days from diagnosis to surgery (95% CI: 0.22–0.53) (Table 9, Figure 10). This finding supports the hypothesis that the timing of genetic testing is associated with the timing of surgery within a plausible clinical window. The observed association reflects shared pathway dynamics: patients with longer overall diagnostic and treatment workflows inherently experienced both later genetic testing and later surgical procedures, consistent with their more locally advanced stage at presentation. Because the longer treatment period is linked to NACT and the no-NACT subgroup in our cohort consists of only 4 patients, as mentioned before, the generalizability of the finding is limited.

### 3.7. Mutation Status and Surgical Timing in the Reimbursement Study Cohort

**Figure 11:**
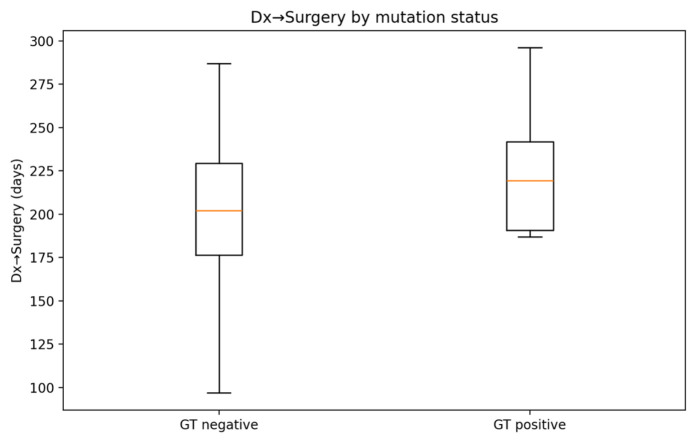
Time from diagnosis to surgery by mutational status (GT—genetic test).

In the reimbursement study cohort (n = 141), mutation-positive patients (n = 18) demonstrated a numerically extended median interval from diagnosis to surgery in comparison to mutation-negative patients (n = 123): 219.5 days (interquartile range 190.8–241.8) versus 202.0 days (interquartile range 176.5–229.5). However, this difference did not attain statistical significance (*p* = 0.168; Cliff’s δ = 0.202) (see Table 10 and Figure 11). In the preoperative testing subgroup, surgical timing did not vary according to mutation status (median 223 vs. 216 days; *p* = 0.982; Cliff’s δ = 0.006) (see Table 11). The observed numerical trend across the entire cohort was entirely absent when the analysis was confined to this clinically pertinent subgroup.

### 3.8. Stratified Analysis: NACT and Genetic Testing Timing Combined in the Reimbursement Study Cohort

A stratified analysis combining NACT status and the timing of genetic testing revealed distinct patterns in clinical pathways (see Table 12). Among patients who received NACT, the interval to surgery was consistently prolonged regardless of the timing of genetic testing (median approximately 210–216 days), thereby emphasizing the predominant role of systemic therapy. Conversely, in the non-NACT pathway, surgery was performed promptly (median approximately 42 days), with the majority of genetic testing conducted postoperatively.

## 4. Discussion

### 4.1. Healthcare System Factors and Testing Integration

The substantially higher preoperative testing rates among the funding programs (PNS 76.7%, PNRR 61%, and private practice 56%) underscore the pivotal influence of healthcare system structure in shaping the quality of genetic testing integration, with no additional delays observed beyond the Neoadjuvant Systemic therapy (NACT) pathways. The superior performance of the PNS program is presumably attributable to established referral pathways, dedicated genetic counseling resources, and standardized testing protocols within the national framework, thereby ensuring access for all patients meeting testing criteria, irrespective of socioeconomic status, recognizing that socioeconomic barriers represented the most significant obstacle to access when subsidized tests were not available. This achievement signifies advancements in the genetic workflow, as dedicated funds enable increased testing volume and more efficient processes. These observations align with existing evidence indicating that mainstreamed and rapid testing programs can notably enhance preoperative testing rates [36,37,38,39].

The elevated rate of NACT in PNS patients (84.5% compared to 73.0% in private practice) partially accounts for the longer interval from diagnosis to surgery in that cohort and should be considered when comparing programs. Notably, the duration from diagnosis to genetic testing did not differ significantly across programs, although turnaround times varied—approximately 64 days from sample receipt to results in the PNRR, primarily due to staffing shortages and limited kits at the onset of testing; a 30-day turnaround in private practice; and approximately 16 days in the PNS, as workflow optimizations were implemented and kits became available and subsidized. This suggests that the primary differentiator lies in the organization of the surgical pathway rather than the speed of the testing process itself.

### 4.2. Principal Findings

This retrospective reimbursement cohort study demonstrates that while genetic testing timing correlates with surgical workflow, NACT pathways and healthcare program structure exert far greater influence on the diagnosis-to-surgery interval than genetic testing per se. NACT emerged as the primary driver of surgical timing, being associated with a median difference of 168 days in diagnosis-to-surgery interval. These findings are consistent with emerging evidence that the NACT window provides a natural and clinically appropriate interval for completing germline testing without compromising surgical timelines, as recommended by international guidelines [24,25,30].

### 4.3. Preoperative Versus Postoperative Testing: Workflow Patterns

The markedly longer interval between diagnosis and surgery in the preoperative testing group (216 versus 182.5 days) reflects the selection of the clinical pathway rather than a causal delay attributable to genetic testing. Patients who proceeded expediently to surgery—primarily those without neoadjuvant systemic therapy (NACT)—were more likely to have genetic testing ordered postoperatively, whereas those undergoing extended NACT pathways had testing integrated preoperatively. This interpretation is corroborated by the stratified analysis, which revealed that within NACT-treated patients, surgical timing remained consistent regardless of the timing of genetic testing (~210–216 days).

These findings are consistent with published data from rapid-testing programs demonstrating that preoperative genetic results can be obtained without causing delays to surgery when appropriate infrastructure is established [36,37]. Yadav et al. [40] reported that preoperative BRCA1/2 testing significantly influenced surgical decision-making, with 95.7% of mutation-positive women utilizing their results to guide surgical choices. Apostolova et al. [25] further showed that preoperative result disclosure increased the uptake of risk-reducing mastectomy and decreased unnecessary exposure to radiotherapy. Roberson et al. [41] emphasized the importance of timely access to testing across diverse populations to prevent disparities in treatment options.

### 4.4. Neoadjuvant Systemic Therapy as an Opportunity for Genetic Testing

The predominant influence of NACT as a determinant of surgical timing (Cliff’s δ = 0.92) highlights a significant clinical opportunity: the neoadjuvant treatment window can represent an important opportunity for completing germline genetic testing in all eligible patients. This strategy is consistent with ESMO guidelines, which recommend offering genetic testing at the time of diagnosis for all eligible patients, regardless of the planned treatment sequence [12,13]. The availability of BRCA mutation status during NACT also permits consideration of platinum-based regimens and the integration of PARP inhibitors in the management of triple-negative breast cancer [7,8,9].

The BrighTNess trial demonstrated that 53.2% of patients initially deemed ineligible for breast-conserving surgery became eligible following neoadjuvant systemic therapy [31]. Consequently, knowledge of BRCA status during NACT can influence not only the surgical approach but also the systemic treatment strategy, thereby underscoring the importance of early genetic testing integration.

### 4.5. Mutation Status and Surgical Decision-Making

The lack of a significant effect of mutation status on surgical timing within the preoperative testing subgroup (*p* = 0.982) is noteworthy. While mutation-positive patients exhibited a numerical trend towards longer intervals in the entire cohort (219.5 versus 202.0 days; *p* = 0.168), this disparity was not statistically significant and was entirely absent within the preoperative subgroup. This indicates that additional surgical planning associated with a positive result—such as consideration of bilateral mastectomy and reconstruction planning—does not substantially extend the time to surgery in contemporary practice [42,43].

The published literature consistently demonstrates that BRCA mutation carriers who receive preoperative results are significantly more likely to undergo bilateral mastectomy at their initial operation [25,40,44,45]. Pederson et al. [46] reported that multigene panel testing influenced surgical decision-making in 24% of patients, with pathogenic variant carriers exhibiting markedly higher rates of bilateral mastectomy. The absence of a timing difference in our cohort suggests that such surgical decisions can be integrated within standard treatment timelines.

### 4.6. Strengths and Limitations

The strengths of this study encompass the extensive, multi-source cohort, the thorough stratification based on NACT status and reimbursement program, and the implementation of robust non-parametric statistical techniques complemented by suitable effect-size measures. The comparison among three distinct funding pathways offers innovative insights into the effect of healthcare system structure on the integration of genetic testing.

Conversely, the limitations include the retrospective nature of the study, which precludes causal inferences. The small number of patients without NACT who underwent preoperative genetic testing (n = 4) restricts definitive conclusions regarding testing-related delays within the fast-track surgical group. Furthermore, the cohort originates from a Romanian institution, and thus, the findings may not be entirely generalizable to healthcare systems with differing testing infrastructure or referral patterns. Additionally, it should be noted that the programs evaluated for efficacy in the genetic testing workflow were implemented sequentially; a potential confounder is the increasing familiarity with the testing process over time and the concurrent enhancement of infrastructure, despite the availability of funding, which we consider validated.

Another limitation of this study is the sample size. A substantial number of potentially eligible patients were excluded because of incomplete data or failure to meet inclusion criteria, which may limit the generalizability of some findings. Because detailed information on excluded patients was not consistently available due to the retrospective design, we could not formally assess whether systematic differences existed between included and excluded individuals. Consequently, selection bias cannot be ruled out and may have influenced the observed results as exclusion of patients with incomplete records could favor cases managed more comprehensively within our institution. The differential inclusion across pathways (36.9% in private practice, 56.3% in PNS, and 65.3% in PNRR), especially affecting the private-practice cohort, suggests differences in the rigor of composing patient files and in access to data across programs, despite the same exclusion criteria. That said, comparisons involving private practice should be interpreted cautiously, as the final analyzable cohort may not fully represent the original private-practice population. Finally, although we controlled for major factors like NACT, additional multivariable considerations or sensitivity analyses could further clarify the independent role of testing timing in surgical delays.

### 4.7. Future Directions

Future research should prospectively assess the influence of rapid or integrated genetic testing on surgical decision-making and patient-reported outcomes across various healthcare environments. Special emphasis should be placed on ensuring equitable access across socioeconomic and geographic divides, as emphasized by Roberson et al. [41] concerning young Black women with breast cancer. Furthermore, the incorporation of multi-gene panel testing and the clinical management of variants of uncertain significance constitute additional domains necessitating prospective investigation.

## 5. Conclusions

This retrospective cohort study suggests that neoadjuvant systemic therapy (NACT) is seen to affect the interval between diagnosis and surgery among breast cancer patients undergoing germline genetic testing. This influence surpasses any independent effect of the timing of genetic testing. Among patients tested prior to surgery, a longer duration to genetic testing was strongly correlated with an extended period before surgery, indicating shared pathway dynamics rather than a causal delay attributable to germline testing. Pathogenic germline variant status did not exert a significant influence on surgical timing within the preoperative subgroup.

Structured reimbursement pathways appear to facilitate better preoperative integration of genetic testing into surgical planning. The national PNS program attained the highest rate of preoperative testing at 78.5%, significantly surpassing PNRR at 61% and self-funded pathways at 53.6%, likely reflecting greater standardization of referral and counseling processes. Importantly, these differences occurred within the broader context of treatment pathway composition, particularly NACT utilization.

These findings endorse the expansion of structured national programs for genetic testing in breast cancer and underscore the neoadjuvant systemic therapy window as an underutilized opportunity for the integration of preoperative testing.

Clinicians and healthcare systems should prioritize the development of rapid or mainstreamed genetic testing pathways that deliver results before surgery, thereby facilitating fully informed surgical decision-making without compromising treatment timelines. National program structures, as exemplified by the PNS experience, can significantly enhance the quality and timeliness of integrating genetic testing in breast cancer care.

Further prospective multicenter studies are warranted to optimize streamlined genetic testing pathways and evaluate their relationship with surgical decision-making in breast cancer care.

## Figures and Tables

**Table 1:** Program Comparison: Preoperative Testing Integration and Surgical Timing for all programs.

Program	Post-Operative Testing (%)	Pre-Operative Testing (%)	Adjusted Pre-Operative Testing Probability	Median Dx→Surgery (Days)	NACT Rate (%)
PNS	21.5	78.5	76.7%	208	84.5
PNRR	39.0	61.0	61.0%	~200	~80
Private practice	46.4	53.6	56.0%	190	73.0

**Table 2:** Logistic regression of the reimbursement programs adjusted for NACT.

Predictor	Adjusted OR	95% CI	*p*-Value
PNRR vs. PNS	0.48	0.19–1.19	0.111
PRIVATE vs. PNS	0.39	0.19–0.80	0.0097
NACT yes	5.56	2.83–10.92	<0.001

OR—odds ratio, CI—confidence intervals.

**Table 3:** Adjusted mean differences Dx→Surgery days in the ordinary least-squares (OLS) linear regression model for all the programs.

Predictor	Adjusted Difference in Dx→Surgery Days	95% CI	*p*-Value
PNRR vs. PNS	−14.9 days	−38.4 to 8.6	0.213
PRIVATE vs. PNS	−28.0 days	−46.5 to −9.6	0.0031
NACT yes	+147.1 days	126.9 to 167.2	<0.001

CI—confidence intervals.

**Table 4:** Adjusted mean differences Dx→Surgery days in the Robust median regression model for all the programs.

Predictor	Adjusted Median Difference in Dx→Surgery Days
PNRR vs. PNS	−13 days
PRIVATE vs. PNS	−14 days
NACT yes	+161 days

**Table 5:** Minimal clinical analysis of the patients in the reimbursement programs cohort.

**Mean age**		52	
**Median age**		51	
**Clinical stage**			
**T**		**N**	
**1**	2%	**0**	9%
**1c**	8%	**1**	43%
**2**	52%	**1a**	3%
**2(m)**	6%	**2**	29%
**3**	11%	**2a**	10%
**3(m)**	2%	**3**	3%
**4b**	19%	**3c**	3%

**Table 6:** Overall Time from Diagnosis to Surgery (n = 141) in the reimbursement study cohort.

Metric	Value	Unit
Median Dx→Surgery	203	days
Interquartile range (IQR)	179–230	days
Mean ± SD	198.3 ± 96.9	days
Range	18–527	days

**Table 7:** Comparison of Surgical Timing: Preoperative vs. Postoperative Genetic Testing in the Reimbursement Study Cohort.

Group	n	Median Dx→Surgery (Days)	IQR (Days)	*p*-Value	Cliff’s δ
GT before surgery day	76	216	197–238	0.000153	−0.486
GT after surgery	28	182.5	42.8–217.2		

**Table 8:** Relationship of Neoadjuvant Systemic Therapy on Time to Surgery.

NACT Status	n	Median Dx→Surgery (Days)	IQR (Days)	*p*-Value	Cliff’s δ
No NACT	25	43	27–69	2.55 × 10^−11^	0.92
NACT yes	116	211	193–232		

**Table 9:** Correlation Between Dx→GT and Dx→Surgery in Preoperative Testing Group (n = 76).

Metric	Value	Interpretation
Spearman ρ	0.54	Moderate positive correlation
*p*-value	4.75 × 10^−7^	Highly significant
Theil–Sen slope	0.37 days surgery per day GT delay	
95% CI for slope	0.22–0.53	

GT—genetic test; Dx—Diagnosis.

**Table 10:** Mutation Status and Surgical Timing: Reimbursement Study Cohort.

Metric	GT Negative (n = 123) Median (IQR)	GT Positive (n = 18) Median (IQR)	*p*-Value	Cliff’s δ
Dx→Surgery (days)	202.0 (176.5–229.5)	219.5 (190.8–241.8)	0.168	0.202
Dx→GT (days)	142.0 (99.5–228.5)	149.0 (72.5–180.0)	0.346	−0.175
GT→Surgery (days)	58.0 (−41.0–110.0)	117.0 (38.5–124.5)	0.097	0.307

Dx—Diagnosis; GT—genetic test.

**Table 11:** Mutation Status and Surgical Timing: Preoperative Testing Subgroup.

Metric	GT Negative (n = 65) Median (IQR)	GT Positive (n = 11) Median (IQR)	*p*-Value	Cliff’s δ
Dx→Surgery (days)	216.0 (197.0–237.0)	223.0 (193.5–256.0)	0.982	0.006
Dx→GT (days)	123.0 (71.0–181.0)	149.0 (72.5–180.0)	0.825	0.043
GT→Surgery (days)	94.0 (49.0–126.0)	117.0 (38.5–124.5)	0.953	−0.013

Dx—Diagnosis; GT—Genetic Test.

**Table 12:** Stratified Timing by NACT Status and Genetic Testing Timing.

Stratum	Dx→GT Median (IQR)	n	GT→Surgery Median (IQR)	n	Dx→Surgery Median (IQR)	n
NACT no + GT pre-op	150.0 (47.0–250.0)	4	36.0 (21.2–63.5)	4	186.0 (81.8–300.0)	4
NACT no + GT post-op	190.0 (113.0–239.0)	13	−160.0 (−224.0–−80.0)	13	42.0 (26.2–49.5)	12
NACT yes + GT pre-op	123.5 (76.2–179.5)	72	95.0 (47.2–130.2)	72	216.0 (197.0–237.2)	72
NACT yes + GT post-op	346.0 (234.0–633.0)	17	−172.0 (−441.0–−45.0)	17	210.0 (191.5–232.0)	16

## Data Availability

The data presented in this study are available on request from the corresponding authors. The data are not publicly available due to privacy and ethical restrictions.

## Abbreviations

The following abbreviations are used in this manuscript:

BRCA1/2	Breast Cancer Gene 1/2
NACT	Neoadjuvant Systemic Therapy
IQR	Interquartile Range
PARP	Poly(ADP-ribose) Polymerase
PNS	Programul Național de Sănătate (National Health Program)
PNRR	Planul Național de Redresare și Reziliență (National Recovery and Resilience Plan)
ESMO	European Society for Medical Oncology
DCIS	Ductal Carcinoma In Situ
VUS	Variant of Uncertain Significance
BSO	Bilateral Salpingo-Oophorectomy
SLND	Sentinel Lymph Node Dissection
ALND	Axillary Lymph Node Dissection
